# Use of Lateral Oncoplasty for Multiquadrant Giant Fibroadenoma: A Novel Approach

**DOI:** 10.7759/cureus.15090

**Published:** 2021-05-18

**Authors:** Sanghamitra Jena, Neetesh K Sinha

**Affiliations:** 1 Surgical Oncology, Tata Main Hospital, Jamshedpur, IND; 2 Surgical Oncology, Medica Cancer Hospital, Rangapani, IND

**Keywords:** giant fibroadenoma, breast, lateral oncoplasty, reconstruction, cosmesis

## Abstract

Giant fibroadenomas are uncommon benign lesions, defined as fibroadenomas of >5 cm in size and/or weighing more than 500 g. They can distort the shape of the breast and cause asymmetry, so they should be excised. Here, we report two cases of giant fibroadenoma, where wide local excision and reconstruction with lateral oncoplasty were done. Compared to all previous reports of patients with giant fibroadenoma, where the lump was excised either through a submammary incision or by round block technique depending on the location of the tumour, we used the lateral oncoplasty technique in both patients. Lateral oncoplasty is a new reconstructive option to maintain cosmesis and symmetry after the excision of giant fibroadenomas in the outer and central quadrants of the breast. It is a good option for reconstruction in cases where the defect is very large and facilities for conventional flap surgeries are not available.

## Introduction

Fibroadenomas in the breast are common benign lesions in women less than 30 years of age. They feature polyclonal proliferation of both epithelial and stromal tissue and are hyperplastic lesions rather than true neoplasms [1]. Giant fibroadenomas, defined as fibroadenomas of greater than 5 cm in size or 500 g in weight, are rare benign breast lesions that account for approximately 0.5%-2% of fibroadenomas [2-4]. We report two cases of giant fibroadenoma treated with a new technique of excision and reconstruction with lateral oncoplasty.

## Case presentation

Case 1

**Figure 1:**
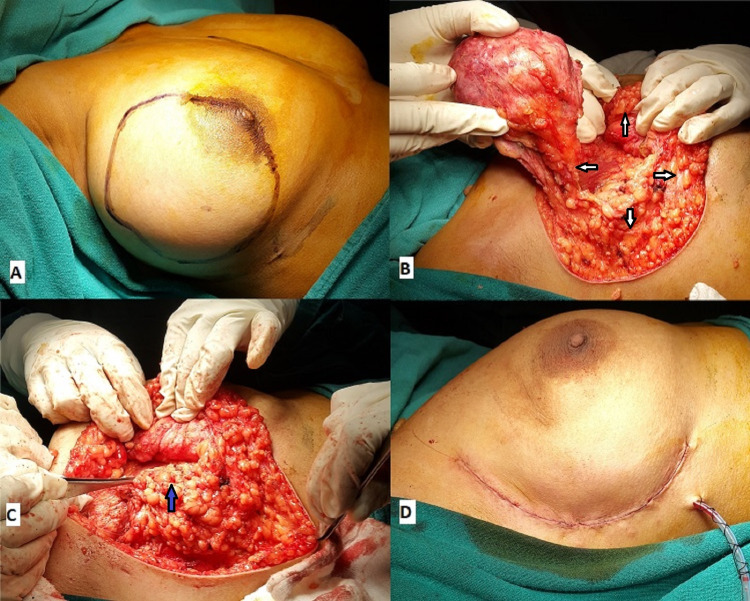
A. Giant fibroadenoma in the outer quadrant of the right breast. B. Well-defined lump excised in total (defect area marked with white arrows). C. Infero-lateral glandular flap raised (marked with blue arrow) and advanced upward and medially. D. Wound closed with subcuticular sutures. Case 1

**Figure 2:**
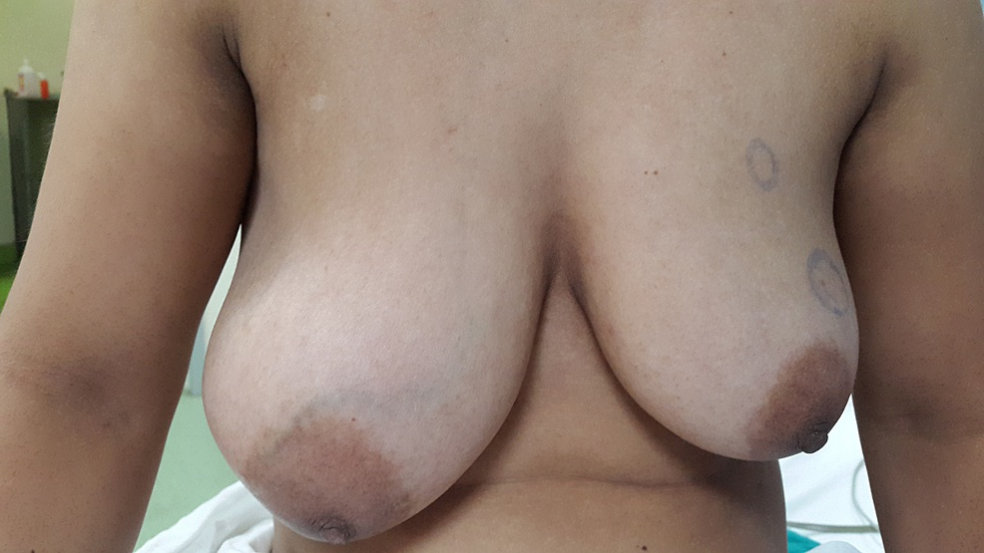
Pre-operative picture of Case 1.

A 25-year-old woman presented with a right breast lump existing for eight months. The mass had rapidly increased in size. Her menstrual cycles were regular and she was not on any hormone therapy. Clinical examination showed a firm and mobile breast tumour on the outer quadrant of the right breast of approximately 10 cm in size, with dilated veins on overlying skin (Figures 1A, 2). There was no axillary lymphadenopathy. Ultrasonography (US) showed a well-defined hypoechoic tumour measuring 10 x 8 cm. Core needle biopsy confirmed fibroadenoma.

The patient underwent right-sided lumpectomy and reconstruction with lateral oncoplasty under general anaesthesia. A skin crease incision was placed in the axillary fold. Subsequently, the skin flap was raised medially over the tumour with a flap thickness of 3-4 mm. The lump was well defined (Figure 1B) and almost the entire normal breast tissue of the affected breast was compressed on to one side by the lump. The lump was completely excised, which left a big defect. An inferolateral glandular flap was raised and advanced upward and medially and fixed with a 2/0 absorbable monofilament suture to the compressed normal breast parenchyma (Figure 1C). A suction drain was left in situ over the pectoral fascia and the wound closed with subcuticular sutures (Figure 1D).

**Figure 3:**
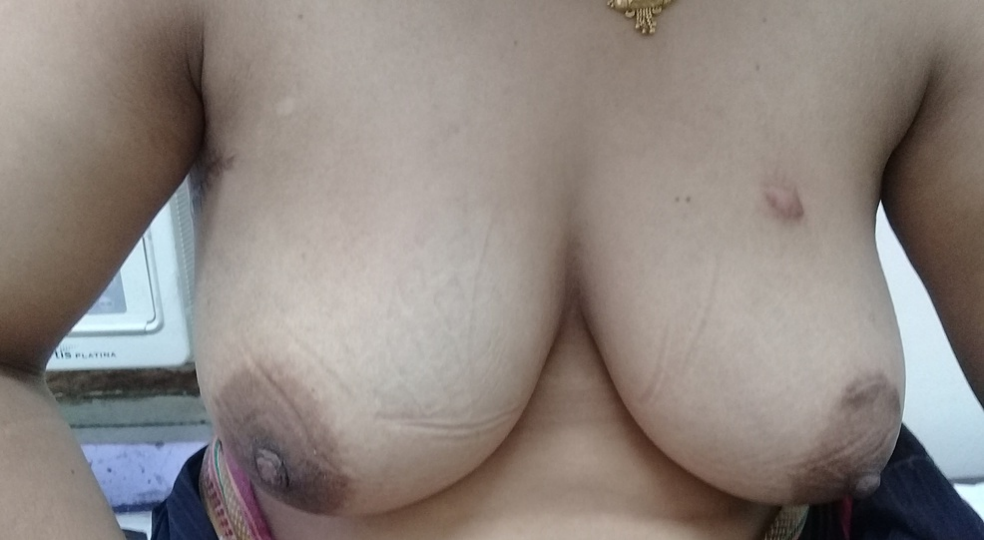
Post operative picture of Case 1 after three months.

The post-operative period was uneventful. The patient was discharged on the third postoperative day after the removal of the drain. The patient was followed up at 10 days, one month, three months, and one year. Complete symmetry was achieved between both the breasts with good cosmesis (Figure 3).

The actual size of the tumour was 10 x 8 x 4 cm. Histopathology showed marked stromal and epithelial proliferation with proliferating stroma around numerous ducts in a peri-canalicular pattern. There was no stromal atypia and no evidence of malignancy. The final diagnosis was reported as giant fibroadenoma.

Case 2

**Figure 4:**
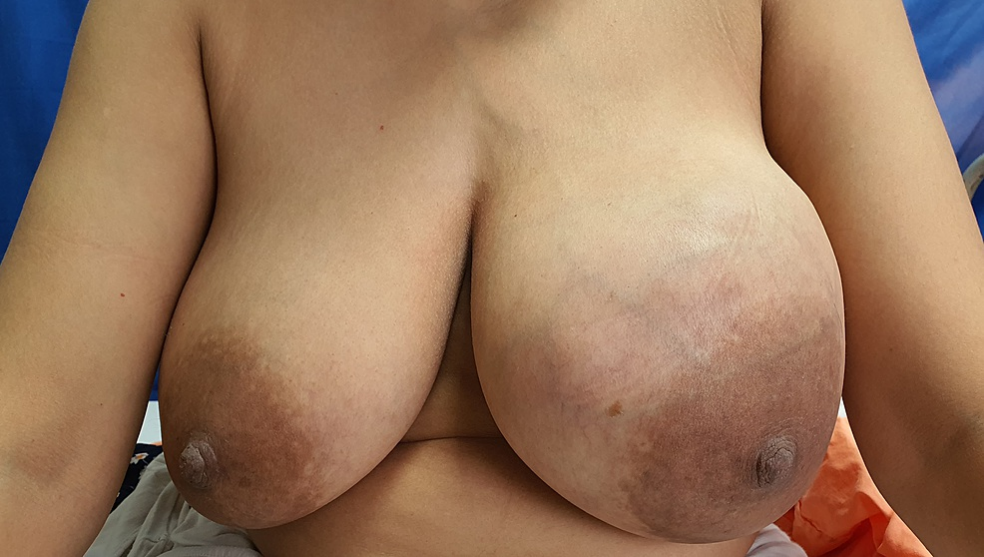
Pre- operative picture of Case 2.

A 28-year-old woman presented with a left breast lump existing for one year. The mass had rapidly increased in size. Clinical examination showed a firm lump in the central quadrant of the left breast of approximately 12 cm in size, with dilated veins on overlying skin (Figure 4). There was no axillary lymphadenopathy. Ultrasonography showed a large well-defined hypoechoic lobulated mass lesion with homogenous echo pattern and internal vascularity in the left-central quadrant measuring 13.8 x 10.5 cm. Core needle biopsy findings were compatible with benign fibro-epithelial lesion favouring benign phyllodes tumour.

**Figure 5:**
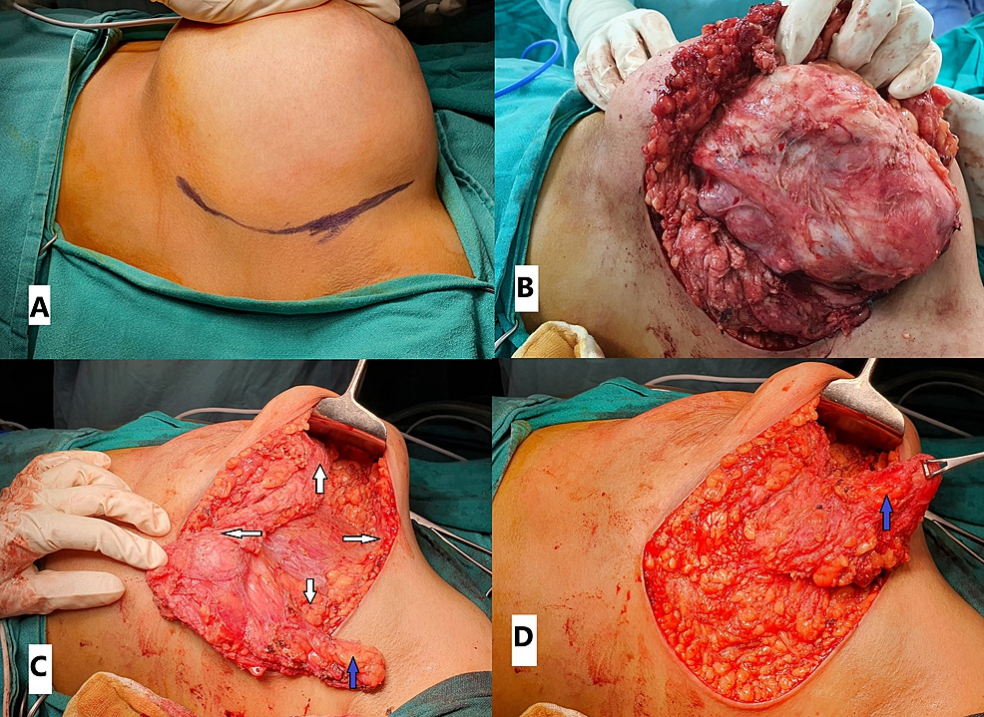
A. Skin crease incision given in lateral axillary fold. B. Well-defined lump. C. Lump excised in total (defect area marked with white arrow and flap marked with blue arrow). D. Lateral oncoplasty done (flap marked with blue arrow). Case 2.

**Figure 6:**
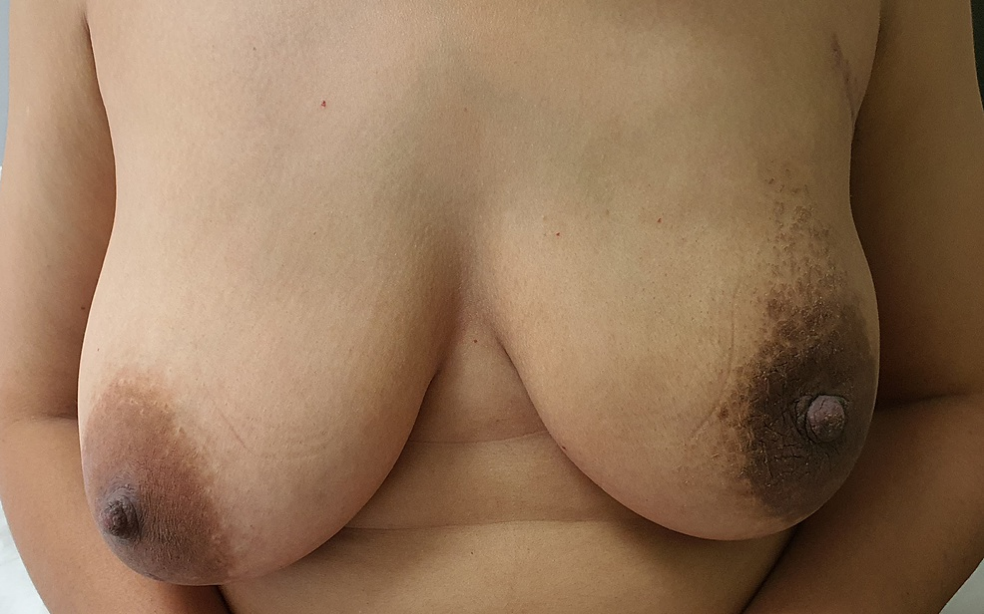
Post operative picture of Case 2 after three months.

The patient was planned for wide local excision of left breast lump and reconstruction with lateral oncoplasty. A skin crease incision was made in lateral axillary fold and flap was raised over the tumour with a thickness of 3-4 mm (Figure 5A). The lump was excised with a margin of 1 cm (Figures 5B, 5C). An inferolateral pedicled dermo-glandular flap was raised and rotated upwards and medially to cover the defect (Figure 5D). A suction drain was placed in situ and wound closed with subcuticular stitches. The drain was removed on the third postoperative day and the patient followed up in OPD on the 10th day, one month, and three months. There was no recurrence, and a good cosmesis was achieved (Figure 6).

The tumour measured 15.5 x 14.5 x 9 cm and weighed 843 g. On cross-section, the lesion was bosselated, nodular with areas of calcification and focal haemorrhage. The final impression was giant fibroadenoma.

## Discussion

Giant fibroadenomas are defined as fibroadenomas of greater than 5 cm in size or 500 g in weight. They are rare benign breast lesions and account for approximately 0.5%-2% of fibroadenomas [2-4]. Both the cases fit well into the criteria of giant fibroadenomas as they are more than 5 cm and weighing more than 500 g. The peak age has been reported between 17 and 20 years [5]. Excessive estrogen stimulation and/or receptor sensitivity or reduced levels of estrogen antagonist during puberty have been implicated in pathogenesis [6,7].

There are a number of differential diagnoses of giant fibroadenomas. In the second case, it was confused with phyllodes tumour. In fact, the clinical and sonographic appearance of giant fibroadenoma is indistinguishable from phyllodes tumour. The characteristics of fibroadenoma include well-demarcated margins, minimal atypia, and rare mitosis while phyllodes tumours sometimes show invasive margins, matrix overgrowth and significant atypia, and a leafy structure [8,9]. Fibroadenomas are frequently hypoechoic with circumscribed margins on US while phyllodes tumors are irregular in shape with heterogeneous internal echogenicity and microlobulated margins. Because the examination of small samples from needle biopsy may yield uncertain results, the final diagnosis is made based on the histopathological report. In the second case, the pathological diagnosis of giant fibroadenoma was made based on the examination of the surgical specimen.

Rapid growth, distortion of breast architecture or with overlying skin changes, and risk of malignancy are indications for surgical excision of a suspected fibroadenoma [4,10]. The goals of surgical reconstruction for giant fibroadenomas with asymmetry should be to achieve equal breast size, reposition the nipple-areola complex, and correct asymmetry with minimal scars in one stage. Multiple treatment modalities ranging from simple excision to mastectomy have been described in the literature [11-13]. The most common incisions reported for excision of giant fibroadenomas are inframammary skin incision, reduction mammaplasty incision, and peri-/circum-areolar incision [1,11,14,15].

In both these cases, a new technique of lateral oncoplasty was used to fill the large defect created after the excision of giant fibroadenoma. This surgical technique was first described for biopsy-proven breast cancer or phyllodes tumour present in the outer quadrant of the breast by Singh et al. [16]. But in our cases, the technique of lateral oncoplasty was used for the reconstruction of tumour involving both outer and central quadrants. In this technique, the breast was dissected of the pectoralis major well beyond the tumor margins. The anterior surface of the breast was separated from the skin, in a plane similar to that while performing mastectomy. Thus, a biplanar mobilization was achieved. The inferolateral glandular flap raised from the inferior quadrant of the breast and lateral axillary fold was then advanced and rotated upward and medially and fixed with 2/0 absorbable monofilament suture with remaining compressed normal breast parenchyma. Here, the upper flap was not needed to achieve a good breast mound and symmetry, as described in Singh et al.'s technique [16]. 

We believe that this approach offers several distinct advantages. There is a single scar in the lateral aspect of the chest wall which remains hidden in the lateral axillary fold. This gives a superior aesthetic result by avoiding any scar over the breast itself. Larger tumors can be excised using this approach as is evident from the size of the tumor and the weight of the resected specimens in both patients. This technique has reduced the use of latissimus dorsi mini flaps for volume replacement in our hands. Therefore, this is not only a more straightforward technique than the current conventional flaps but also a technique with short operation time and less postoperative pain or other morbidities to patients.

The complications associated with this technique are surgical site infections, delayed wound healing, marginal skin necrosis, superficial NAC (nipple-areola complex) necrosis, and fat necrosis [16]. But in our cases, there was no complication postoperatively. The surgical site infection which occurs most commonly can be managed according to the American Society of Breast Surgeons consensus guideline on preoperative antibiotics and surgical site infections (SSI) in breast surgery [17]. The skin margin necrosis and NAC necrosis can be managed surgically by debridement and margin revision. The fat necrosis can be managed conservatively or with debridement.

## Conclusions

Giant fibroadenoma causes asymmetry in both breasts and requires wide local excision. This causes a large defect after excision. Lateral oncoplasty is a new reconstructive option to maintain cosmesis and symmetry after the excision of giant fibroadenomas in the outer and central quadrants of the breast. This procedure may be worth considering as an option for reconstruction after the excision of giant fibroadenoma in cases where the defect is large and facilities for conventional flap surgeries are not available.
